# Association between Vitamin D Deficiency and Single Nucleotide Polymorphisms in the Vitamin D Receptor and GC Genes and Analysis of Their Distribution in Mexican Postmenopausal Women

**DOI:** 10.3390/nu10091175

**Published:** 2018-08-27

**Authors:** Berenice Rivera-Paredez, Nayeli Macías, Mayeli M. Martínez-Aguilar, Alberto Hidalgo-Bravo, Mario Flores, Amado D. Quezada-Sánchez, Edgar Denova-Gutiérrez, Miguel Cid, Angelica Martínez-Hernández, Lorena Orozco, Manuel Quiterio, Yvonne N. Flores, Jorge Salmerón, Rafael Velázquez-Cruz

**Affiliations:** 1 Academic Unit in Epidemiological Research, Research Center in Policies, Population and Health, School of Medicine, National Autonomous University of Mexico, Mexico City 04510, Mexico; bereriveraparedez7@gmail.com (B.R.-P.); jorge.salmec@gmail.com (J.S.); 2 Nutrition and Health Research Center, National Institute of Public Health (INSP), Cuernavaca, Morelos 62100, Mexico; nmacias@insp.mx (N.M.); mario.flores@insp.mx (M.F.); edenovag@gmail.com (E.D.-G.); 3 Genomics of Bone Metabolism Laboratory, National Institute of Genomic Medicine (INMEGEN), Mexico City 14610, Mexico; mmaye.sol@gmail.com; 4 Department of Genetics, National Institute of Rehabilitation (INR), Mexico City 14389, Mexico; dr_genetica@yahoo.com; 5 Center for Evaluation and Surveys Research, National Institute of Public Health (INSP), Cuernavaca, Morelos 62100, Mexico; amado.quezada@insp.mx; 6 Inmunogenomics and Metabolic Diseases Laboratory, National Institute of Genomic Medicine (INMEGEN), Mexico City 14610, Mexico; macs090883@yahoo.com.mx (M.C.); amartinez@inmegen.gob.mx (A.M.-H.); lorozco@inmegen.gob.mx (L.O.); 7 Center for Population Health Research, National Institute of Public Health (INSP), Cuernavaca, Morelos 62100, Mexico; mquitero@insp.mx; 8 Epidemiology and Health Services Research Unit, Mexican Institute of Social Security, Cuernavaca, Morelos 62000, Mexico; ynflores@ucla.edu; 9 UCLA Department of Health Policy and Management, UCLA Kaiser Permanente Center for Health Equity, Fielding School of Public Health and Jonsson Comprehensive Cancer Center, Los Angeles, CA 90001, USA

**Keywords:** total 25(OH) vitamin D, genetic polymorphism, vitamin D-binding protein, vitamin D receptor, bone mineral density, Mexican-Mestizo

## Abstract

Genome-wide association studies in people with European ancestry suggest that polymorphisms in genes involved in vitamin D (VD) metabolism have an effect on serum concentrations of 25-hydroxyvitamin D. However, nothing is known about these polymorphisms in populations with Amerindian ancestry. Our aim was to evaluate the association between genetic variants on the vitamin D receptor (*VDR*) and the vitamin D binding protein (*GC*) genes, involved in the VD pathway, and VD deficiency in 689 unrelated Mexican postmenopausal women. We also described the frequencies of these variants in 355 postmenopausal women from different ethnic groups. Based on our preliminary results of 400 unrelated Mexican postmenopausal women, three single nucleotide polymorphisms (SNPs) were selected for genotyping. The SNPs rs4516035 in *VDR* and rs2282679 in *GC* were associated with VD deficiency. Additionally, women who carried three risk alleles had a 3.67 times higher risk of suffering VD deficiency, compared to women with no risk alleles (*p* = 0.002). The rs4516035-C allele frequency in the Amerindian population was enriched in the South East region of Mexico. In contrast, the highest frequency of the rs2298850-C allele, a proxy for the tag SNP rs2282679, was observed in the South region. Our results indicate that genetic variants in *VDR* and *GC* genes are associated with VD deficiency in Mexican postmenopausal women. Moreover, an association was observed for the variants rs3794060 and rs4944957 of the *DHCR7/NADSYN1* gene with osteopenia/osteoporosis.

## 1. Introduction

Mexico is undergoing demographic changes characterized by a growing number of people over the age of 50 years. The 2015 population census reported an estimated 23 million people within this age group. According to The National Population Council of Mexico (CONAPO), the proportion of individuals over 50 years of age will increase more than 55% by 2020, and up to 200% by 2050 [1]. The aging of the population, accompanied by an extended life expectancy, may result in a higher prevalence of osteopenia and osteoporosis. In Mexico, the estimated prevalence of osteopenia and osteoporosis is 56% and 16%, respectively [2]. Furthermore, osteoporosis increases fragility fractures, resulting in higher morbidity, mortality and economic burden, which is a growing public health problem [2]. Osteoporosis also causes approximately 30,000 femur and/or hip fractures per year and accounts for 19.5% of vertebral fractures in women [3].

Vitamin D (VD) regulates calcium (Ca) and the intestinal absorption of phosphorous. As part of the aging process, serum parathyroid hormone increases and serum 25-hydroxyvitamin D (25(OH) D) diminishes [4,5]. Low serum concentrations of 25(OH)D are associated with low bone mineral density (BMD) [4,5]. A reduction of BMD can lead to osteopenia and osteoporosis; therefore, detection of 25(OH)D deficiency should be considered a public health priority. Globally, an estimated 1 billion people have some form of VD deficiency [6]. In Mexico, the prevalence of VD deficiency ranges from 8% to 24% in all age groups. However, among post-menopausal women, the prevalence has been reported to be as high as 46.7% [7,8,9]. The main source of 25(OH)D is 7-dehydrocholesterol which is activated by sunlight ultraviolet B (UVB) radiation. In order to become its active molecule, 7-dehydrocholesterol goes through hydroxylation, first in the liver and then in the kidneys [10]. The main food sources of VD are milk, oily fish and fortified foods. Some factors that can affect VD concentrations are age, body adiposity, race, and genetic variants [11]. Genetic factors have been found to contribute to VD concentration by as much as 53% [12]. The genetic variants derived from genome-wide association studies (GWAS) conducted on European populations that are associated with VD concentrations are engaged in cholesterol synthesis (*NADSYN1/DHCR7* gene), hydroxylation (*CYP2R1* gene) and VD transport (*GC* gene) [13,14]. To the best of our knowledge, there are no studies that have evaluated the relationship between VD metabolism and its deficiency with the genetic variants of the above-mentioned genes in Mexican population. The aim of the present study was to evaluate the association between genetic variants in the genes involved in the VD pathway and VD deficiency in Mexican postmenopausal women. In addition, the frequencies of these variants are described in Mexican Amerindian ethnic groups.

## 2. Materials and Methods

### 2.1. Participants—Second Wave of the Health Workers Cohort Study

The present study is a cross-sectional analysis. The data was collected during a second assessment of the “The Health Workers Cohort Study” (HWCS) from 2010–2012. The HWCS is a prospective study conducted in workers from the Mexican Institute of Social Security (IMSS) in Cuernavaca, Morelos (located in the central region of Mexico). The HWCS focuses on the association between different health endpoints with genetic background and lifestyle. A total of 1855 HWCS participants aged 18 to 85 years were considered for the second evaluation. The variables and procedures of the HWCS have been described in detail somewhere else [15]. We analyzed the SNPs and VD concentrations of 689 out of 754 postmenopausal women evaluated during 2010–2012 (Figure S1). We excluded a total of 65 women; 35 related women, 27 without SNP quality control and three women with missing 25(OH)D concentrations. In order to know the frequencies of the genetic variants of interest in Mexican Amerindian populations, we evaluated an independent sample of 355 postmenopausal women from 37 different ethnic groups taken from the Metabolic Analysis in an Indigenous Sample (MAIS) cohort [16]. Women from the MAIS cohort were included according to the following criteria: (1) women who identified themselves as indigenous, (2) her parents and grandparents spoke the same language and (3) they were born in the same area as their parents and grandparents. The Mexican population is highly heterogenic and according to previous data, there is a gradient of European ancestry from North to South [16,17,18]. Based on this, the Amerindian populations were classified into five geographic regions: North (N), Central East (CE), Central West (CW), South (S) and Southeast (SE) [16]. This study was planned and performed according to the guidelines of the Declaration of Helsinki. The Research Ethics Committee of the IMSS (No. 12CEI 09 006 14) approved the study protocol and informed consent forms. Written informed consent was obtained from all participants.

### 2.2. Data Collection and Blood Sample Collection

Participants answered a self-administered questionnaire focused on characteristics such as birth date, education, marital status, medical family history, past medical history, current medication use, lifestyle information (e.g., diet, physical activity, smoking status, alcohol consumption). Anthropometric body composition measurements and blood samples were collected using standard procedures. Physical activity information was obtained with a questionnaire validated in Spanish in a sample with similar characteristics [19,20]. Participants reported recreational physical activity such as walking, running, etc. during a typical week of the previous year.

### 2.3. Anthropometric Measurements

Body weight was measured with a calibrated electronic scale (model BC-533, Tanita, IL, USA) with participants wearing underwear and bare feet. Height was taken using a conventional stadiometer (Seca HH, Hamburg, Germany), participants were standing barefoot with relaxed shoulders. Height was taken while touching the top of the head at the moment of maximum inspiration. Waist circumference was measured at the highest point of the iliac crest [21]. All measurements had concordance coefficients between 0.83 and 0.90. All measurements were performed by trained personnel using standardized techniques [21].

### 2.4. Body Composition Measurements

Bone mineral density (BMD), bone mineral content, lean body mass, appendicular lean body mass, and fat mass, were obtained with a Lunar DPX NT instrument (Lunar Radiation Corp., Madison, WI, USA) with standardized procedures and trained personnel [22]. Briefly, daily quality assurance scans were conducted using the phantom provided by the manufacturer; the daily variation coefficient was within normal operational standards and the in vivo variation coefficient was lower than 1.0%–1.5% [15]. Women attended the body composition evaluation after 12 h of fasting, neither drinking alcohol nor exercising. A dual-energy X-ray absorptiometry (DEXA) measurement was performed at the lumbar spine (L2–L4), hip, femoral neck, Ward’s angle and total body, to obtain density values expressed as g/cm^2^. We used World Health Organization criteria, T-score, to classify women with osteopenia or osteoporosis. A T-score > −1 standard deviation (SD) was considered as normal, from −1.0 to −2.5 SD as osteopenia, and under −2.5 SD as osteoporosis [23].

### 2.5. Dietary Measurements

We used a semi-quantitative food frequency questionnaire, previously validated in a Mexican population [24]. The questionnaire collects data about the frequency of consumption of 116 food items during the previous year. The instrument specifies commonly used size portions. We calculated the energy and nutrient intake by multiplying the frequency of consumption of each food by the nutrient content. We took the information from a comprehensive database of food contents [25].

### 2.6. Biological Measurements

Blood samples, approximately 20 mL, were taken after 8 h of fasting. We evaluated aminotransferase, triglycerides, glucose, total cholesterol, high-density lipoproteins, low-density lipoproteins, uric acid, creatinine, etc. All biomedical assays were performed using a Selectra XL instrument (Randox Laboratories Ltd., Antrim, UK), in concordance with the proceedings of the International Federation of Clinical Chemistry and Laboratory Medicine [26]. Genomic DNA was isolated from peripheral blood leukocytes using the Gentra Puregene Blood Kit (QIAGEN, Minneapolis, MN, USA), according to the manufacturer’s instructions.

### 2.7. Measurement of Plasma 25(OH)D

Serum 25-hydroxyvitamin D was measured with an Abbott Architect^®^ Chemiluminescent Microparticle Immuno Assay (CMIA, Lake Bluff, IL, USA). The intra- and inter-assay variation coefficients of the method have been reported as <10%. This method has shown high reactivity (~100%) with 25-hydroxyvitamin D3 and an acceptable performance compared to LC/MS/MS (*r* = 0.73) [27]. Chemiluminescence has been reported as an accurate and reproducible method [28]. VD deficiency was defined as serum 25(OH)D levels <20 ng/mL, as reported in a previous study in an elderly Mexican population [9].

### 2.8. Single Nucleotide Polymorphisms Selection and Genotyping

Single nucleotide polymorphisms (SNPs) identified through genome-wide association studies of 25(OH)D serum concentrations and previous association studies in European populations were considered for inclusion. Twenty-nine SNPs involved in the VD metabolic pathway were selected (Table S1) [11,29,30]. In the first stage, a GoldenGate design (Illumina, San Diego, CA, USA) with a total of 384 SNPs (including the 29 SNPs within the VD metabolic pathway) was performed on 400 postmenopausal women from the HWCS. The information obtained was used to test associations between the SNPs and BMD. Information of the parental frequencies and genotype data from a panel of 96 Ancestry Informative Markers (AIMs) was included in the analysis. This information was included in order to control for the effect of false associations due to population stratification. Genotype data of the 29 SNPs was extracted from the initial study for further analysis. The primary analysis of the genotyping data was carried out using the Illumina GenomeStudio software v.2011.1 (Illumina, Inc., San Diego, CA, USA), as described previously [31]. Based on this analysis, the SNPs rs10783219 and rs4516035 on the *VDR* gene and rs2282679 on the *GC* gene were selected, considering the statistical significance (*p* < 0.05) and that they were not in linkage disequilibrium, for further genotyping in the remaining 289 postmenopausal women. The SNPs were genotyped using commercial predesigned TaqMan Probes (Applied Biosystems, Foster City, CA, USA) and called automatically using the SDS 2.2.1 software (Applied Biosystems, Foster City, CA, USA). In both stages, quality control exclusions were implemented because of low SNP call rate (<97%), deviation from the Hardy–Weinberg equilibrium (*p* ≤ 0.05), related women and gender concordance. After quality control, 689 postmenopausal women with both genetic data and 25(OH)D measurements were available for analysis. The MAIS cohort samples, were previously genotyped with the Genome-Wide Human SNP 6.0 Microarray (Affymetrix, Santa Clara, CA, USA) [16].

### 2.9. Construction of the Genetic Risk Score (GRS)

To evaluate the combined effect of the SNPs that were significantly associated with vitamin D deficiency, we constructed a genetic risk score (GRS) [32] for each individual, which included two SNPs (rs2282679-GC and rs4516035-VDR). The GRS (ranging from 0 to 4) was constructed by summing the number of risk alleles from these two SNPs for each individual. Genotypes for each SNP were scored using an additive model (0 for homozygous for the non-risk allele, 1 for heterozygous, and 2 for homozygous for the risk allele).

### 2.10. Statistical Analysis

Samples were grouped according to VD serum concentrations. A *t*-test and chi-square test were used for analyzing sociodemographic and clinical variables between subjects with deficient and normal concentrations of VD. Genotype and allele frequencies were calculated for every SNP. The Hardy-Weinberg equilibrium was tested for each SNP using a chi-square test. A multiple logistic regression model was fit using vitamin D deficiency as the dependent variable; all the independent variables were simultaneously included. The association between SNPs or GRS and VD deficiency was evaluated through logistic regression models adjusted by co-variables of interest in order to obtain odds ratio (ORs) and 95% confidence intervals (95% CI). The first model was adjusted by age (<60, 60−74, >74 years), body mass index (normal <25 kg/m^2^, overweight 25 to <30 kg/m^2^, obesity ≥30 kg/m^2^), VD intake (tertiles), season of the year when the blood sample was collected (spring, summer, winter, autumn) and physical activity (active ≥30 min/day). A second model using body fat percentage instead of body mass index (BMI) was generated. However, because the estimators did not change, only the second model, adjusted by body fat percent, is presented. A plot of the prevalence of vitamin D deficiency or OR (CI 95%) and GRS was generated. In addition, a sensitivity analysis was performed using a linear regression model to evaluate the association between serum 25-(OH) D levels and the SNPs of the *GC* and *VDR* genes; adjusting for co-variables. To approximate normality in vitamin D levels, a square root transformation was performed, as described in Xu et al. [33]. The association between SNPs and BMD was evaluated through logistic regression adjusted by age and BMI. In addition, VD concentrations (non-deficient and deficient), blood collection season (winter, spring, summer and autumn) and physical activity (active ≥30 min/day) were included in a second logistic regression model. A value of *p* < 0.05 was considered to be statistically significant. All statistical tests were performed with STATA 13.0 (Stata Corp, College Station, TX, US) for Windows [34]. We used the HaploView 4.1 software (Broad Institute, Cambridge, MA, USA) to evaluate the linkage disequilibrium [35]. Allelic frequency distribution in Mexican Amerindian (MA) populations was represented on maps taken from the National Commission of Knowledge and Use of Biodiversity (CONABIO). Maps were analyzed using the software R and QGIS 2.14 (Quantum Geographical Information Systems) [16]. To allow for multiple testing (3 SNPs), we report the results of a Bonferroni-corrected *p* value of 0.02.

## 3. Results

### 3.1. Participant Characteristics

The study included a total of 689 non-related postmenopausal women between 45–92 years. The average age was 62 years (SD ± 8.8), more than 45% were overweight and around 30% were obese. Average serum concentration of VD was 21.0 ng/mL and mean VD intake was 199 UI/day. The prevalence of VD deficiency was near 44%. The most frequent chronic diseases in the study population were hypertension (45%), osteopenia/osteoporosis (38%) and hyperuricemia (33%) (Table 1).

There was a positive association between VD status and age; VD-deficient women were older, had a lower mean BMD of the femoral neck and spine and had a higher prevalence of diabetes in comparison to women without VD deficiency (*p* < 0.05). Moreover, VD deficient-women had a lower average of VD intake in comparison to women with no deficiency (*p* < 0.05) (Table 2).

### 3.2. Factors Asociated with Vitamin D Deficiency

Logistic regression analysis was adjusted for education, smoking, VD intake, season of blood collection, leisure time physical activity and body mass index. Results revealed that women between 60 and 74 years and women older than 74 years old had an OR of 1.95 (95% CI 1.36−2.79) and 3.07 (95% CI 1.70−5.56), respectively of having VD deficiency compared to woman between 45–59 years old. Additionally, it was observed that women with the highest intake of VD had a 39% (*OR* = 0.61, 95% CI: 0.39−0.95) lower probability of presenting with VD deficiency in comparison to women in the lowest tertile of VD intake. Results were adjusted by the same variables mentioned above (Table S2).

### 3.3. Association between SNPs and Vitamin D Deficiency

In the first stage, we identified five SNPs lying on the *GC* gene, which is involved in VD transport. We also identify three SNPs on the vitamin D receptor gene (*VDR*) that were positively associated to VD deficiency (Table S3). This result was conserved after excluding 29 women with a VD serum concentration beyond 29 ng/mL; for example, the SNP rs4516035 had an *OR* of 3.50 (95% CI: 1.37−8.89) for VD deficiency (data not shown). The results of crude and adjusted analysis for the VDR variants were statistically significant. We observed an increased risk for VD deficiency with the variants rs10783219, rs4516035 and rs7139166 under co-dominant and additive genetic models (Table S3).

The association for the *GC* gene variants (rs17467825, rs2282679, rs3755967, rs2298850 and rs1155563), under an additive model, was statistically significant in both the crude and adjusted models. For the SNPs rs17467825, rs2282679 and rs3755967, the associations with VD deficiency remained statistically significant for the risk genotype under a co-dominant model. However, for the rs2298850 and rs1155563 variants, the association did not remain statistically significant under the co-dominant model. The SNPs rs7041 and rs12512631 had an inverse association with VD deficiency (*p* = 0.03), only with the unadjusted model. The polymorphisms associated with VD deficiency had strong linkage disequilibrium. The SNPs rs17467825, rs2282679, rs3755967, and rs2298850 of the *GC* gene had an *r^2^* value of >0.93 and for the VDR SNPs, rs4516035 and rs7139166 had an *r^2^* value of 0.95 (Figure S2). Two SNPs were chosen as the tagging SNPs (rs4516035 and rs2282679).

In the second stage, the SNPs rs10783219, rs4516035 and rs2282679 were selected to be evaluated in the entire postmenopausal women cohort. For SNPs rs4516035 and rs2282679, under the additive model, it was observed that the OR increased with each additional copy of the minor allele (*OR* = 1.40, 95% CI: 1.08−4.43 and *OR* = 1.53, 95% CI: 1.15−2.04, respectively). However, for the SNP rs10783219, the association was no longer statistically significant (*p* = 0.377) (Table 3). Additionally, we evaluated the serum 25-(OH)D levels and the SNPs of the *GC* and *VDR* genes. However, only the SNP rs2282679 (*β* = −0.12 (95% CI: −0.20, 0.04), *p* = 0.004) of the *GC* gene was found to be associated with serum 25(OH)D levels under the additive model (data not shown).

### 3.4. Association between GRS and Vitamin D Deficiency

**Figure 1:**
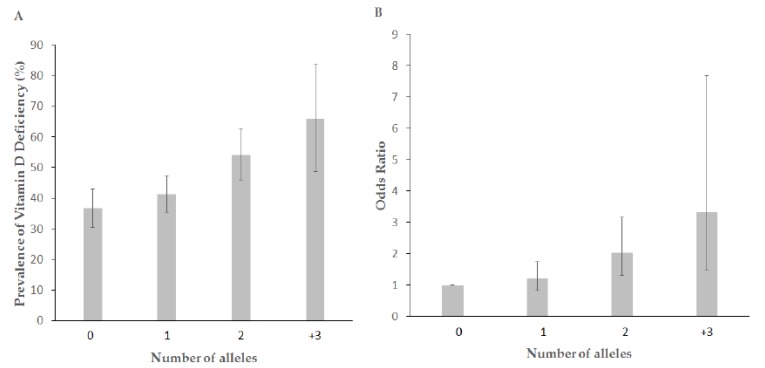
Dose-response relationship between the number of risk alleles in rs4516035, rs2282679 and vitamin D deficiency. * Lines indicate 95% confidence intervals. (**A**) Prevalence of vitamin D deficiency status by GRS. (**B**) Odds Ratio of vitamin D deficiency status by GRS.

The results of the genetic risk score generated with the combination of two variants showed that the greater the number of risk alleles, the higher the VD deficiency prevalence. For individuals with no risk alleles, the prevalence of VD deficiency was 36.8 compared to 66.1 for individuals with three or more risk alleles (*p* = 0.002) (Figure 1A). In addition, the genetic risk score (combining the two variants) was associated with the risk of VD deficiency, with an adjusted OR in the top category of 3.34 (95% CI: 1.46–7.68, *p* = 0.004) (Figure 1B).

### 3.5. Association between SNPs and Bone Mineral Density

We also explored whether the variants have some relationship with bone mineral density (BMD). A statistically significant association was observed for the variants rs3794060 and rs4944957 of the *DHCR7/NADSYN1* gene with osteopenia/osteoporosis. After adjusting for age and body mass index, the association persisted for the two variants (*p* < 0.05) (Table S4). Additionally, the model was adjusted for VD concentrations, blood collection season and physical activity, and the association was not affected (*p* < 0.05) (data not shown).

### 3.6. Minor Allele Frequency

Table S1 shows the minor allele frequency (MAF) distribution between Utah residents with Northern and Eastern European ancestry (CEU), Los Angeles residents with Mexican ancestry (MXL), and the Mexican cohort HWCS. MAFs of the tagging SNPs rs4516035 (on the *VDR* gene) and rs2282679 (on the *GC* gene) were compared between CEU, MXL and HWCS populations. The “C” allele of the rs4516035 SNP was more frequent in European populations (0.37), while the frequency in the HWCS cohort was very similar to the frequency observed in the population with Mexican ancestry from Los Angeles (0.267 versus 0.27). With respect to the frequency of the “C” allele of rs2298850, which is a proxy for rs2282679, it was more frequent in residents from Los Angeles with Mexican ancestry (0.27); the frequency in the HWCS cohort was similar to the frequency observed in European populations (0.23 in both) (Table S1).

### 3.7. Minor Allele Frequency from Different Ethnic Groups

**Figure 2:**
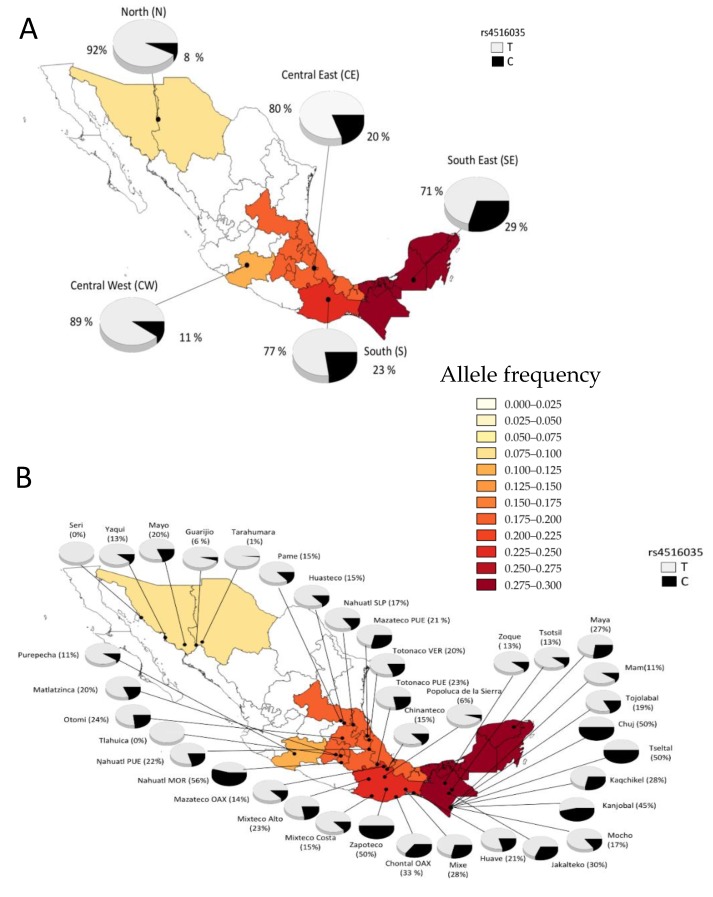
Geographic distribution of the allelic frequencies (T/C) for the rs4516035 polymorphism in Mexican Amerindian groups. MEX, Mexico State; MOR, Morelos; OAX, Oaxaca; PUE, Puebla; SLP, San Luis Potosi, VER, Veracruz. (**A**) Distribution of the allelic frequencies for the rs4516035 polymorphism within each geographic region. (**B**) Allelic frequencies for the rs4516035 polymorphism in the Amerindian groups.

**Figure 3:**
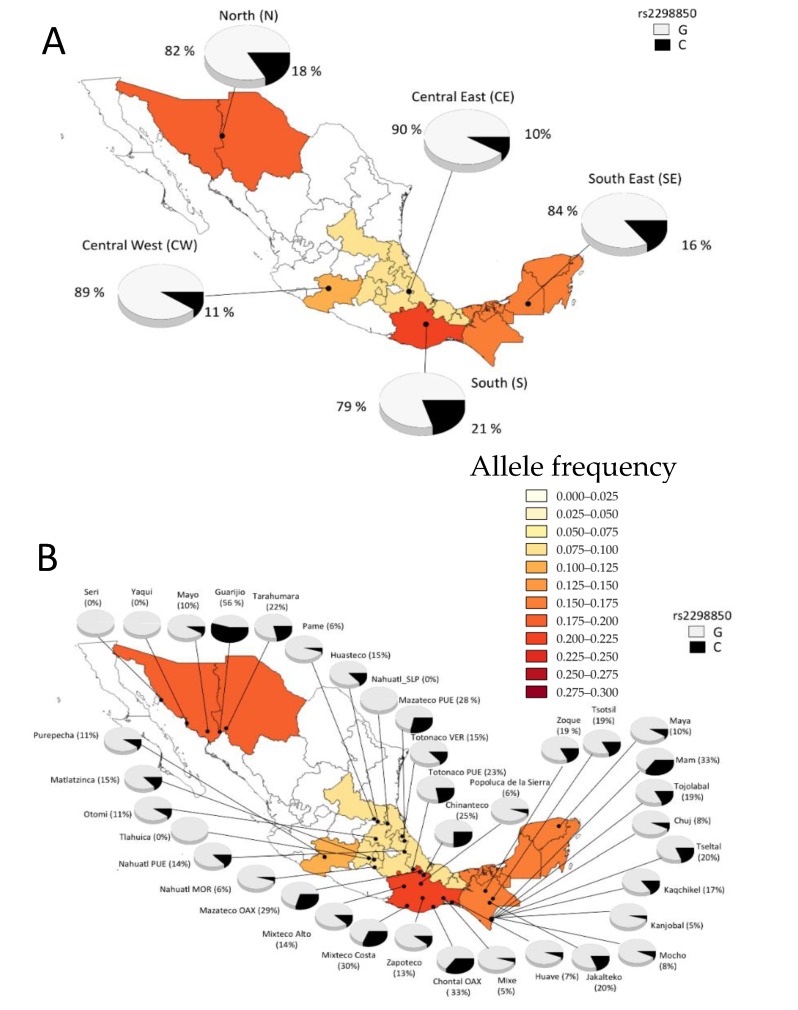
Geographic distributions of the allelic frequencies (G/C) for the rs2298850 polymorphism, (proxy for rs2282679) in the Mexican Amerindian groups. MEX, State of Mexico; MOR, Morelos; OAX, Oaxaca; PUE, Puebla; SLP, San Luis Potosi, VER, Veracruz. (**A**) Distribution of the allelic frequencies for the rs2298850 polymorphism within each geographic region. (**B**) Allelic frequencies for the rs2298850 polymorphism in the Amerindian groups.

To obtain a global view of the frequency of the rs4516035 and rs2298850 SNPs in the Mexican population, we were able to evaluate the allele and genotype frequencies in 37 different Mexican Amerindian groups (Table 4). The genotype distribution for the two SNPs evaluated was in Hardy–Weinberg equilibrium in Mexican Amerindian populations. Ancestry analysis showed an average Amerindian ancestry of 95 ± 5.7% in the MAIS sample [16]. The 37 Mexican Amerindian (MA) groups were sorted into five major geographic regions (North (N), Central East (CE), Central West (CW), South (S) and South East (SE)). This analysis showed a high heterogeneity of the allele and genotype frequencies for both polymorphisms between regions. Frequencies followed a geographic gradient across Mexico, similar to previous reports [36]. The frequency of the rs4516035-C allele was enriched in the SE region (29%), followed by the S (23%) and CE regions (20%). The lowest frequency was found in the N region (8%) (Figure 2A). The rs4516035-C allele showed the lowest frequency in the N (0–20%) and in a few groups from the other geographic regions, such as Tlahuicas (CE) and Popolucas from the Sierra (CE). Of note, the *CC* genotype was absent in groups from the N and CW regions, and was only present in a few groups such as Nahuatls from Morelos (CE); Chontals from Oaxaca, Huaves and Mixtecos from the Costa (S); Chujs, Kanjobals, Kaqchikel, Mayas, Mochos and Tseltals (SE) (Table 4 and Figure 2B). Notably, the highest frequencies of the rs4516035-C allele were observed among Nahuatls from Morelos (56%) in the CE region; Zapotecos (50%) and Chontals from Oaxaca (33%) in the S region; Tseltals (50%), Kanjobals (45%), Jakaltecos (30%) and Kaqchikels (28%), inhabiting the SE (Table 4 and Figure 2B). The geographic distribution of the rs2298850-C allele frequency, the proxy for rs2282679 (the tag SNP), followed a gradient opposite to that observed for rs4516035 (Figure 3). In general, frequencies were very similar through geographic areas: N (18%), CW (11%), CE (10%) and SE (16%), with the exception of the S region that showed the highest frequency (21%) (Table 4 and Figure 3A). The *CC* genotype was only present in a few groups, such as Guarijio (N), Matlaltzinca (CE); Chontal and Mazateco from Oaxaca, and Mixteco from the Costa in the S region and Mam (SE) (Table 4). The highest frequencies of the rs2298850-C allele were found in the Guarijío (56%), Mixteco-Costa (30%), Chontal from Oaxaca (33%) and Mam (33%) (Figure 3B).

## 4. Discussion

The aim of this study was to evaluate the association between SNPs lying on genes involved in VD metabolism with vitamin D deficiency in a sample of Mexican postmenopausal women. We identify two genetic variants of genes participating in the VD metabolic pathway associated with VD deficiency. Under an additive model, we observed a 40% increased risk of suffering VD deficiency for each risk allele of the rs4516035 SNP. Similarly, the presence of two risk alleles of *GC*, the rs2282679 SNP, was highly associated with VD deficiency. Furthermore, as the number of alleles increased, there was a higher risk of having VD deficiency.

VD deficiency was highly prevalent in our study at almost forty percent. This prevalence is similar to that reported by the Nutrition and Health National Survey in Mexico performed in 2012 [37]. Other studies have shown similar findings; in a sample of Mexican elderly women, there was a prevalence of VD deficiency of 46.8%, compared with the prevalence of 63.0% in Hispanic women and 29.5% in women with osteoporosis [9,38,39].

It has been documented that age, BMI and VD intake are the main variables associated with VD deficiency [9,40,41]. We observed a higher frequency of obesity in VD deficient women compared to the group with no deficiency, however, this difference was not statistically significant. A previous study in the Mexican elderly population observed that for each unit of BMI increment, the OR for suffering VD deficiency increased 1.03 times (95% CI: 1.01–1.06) [9]. Additionally, a study in older Mexican adults showed that overweight/obese individuals had 1.78 times risk of having VD concentrations within the first tertile (≤20.4 ng/mL) and 1.94 times within second tertile (20.5–26.6 ng/mL), compared to individuals with normal weight [41]. Although a relationship between BMI and VD status has been documented, some studies have not found such association. For example, Pathak et al., did not find an effect of VD supplementation on adiposity in their meta-analysis. Thus, the association between adiposity and VD remains controversial [42]. A possible explanation for not finding an association between BMI and VD could be the genetic heterogeneity of the Mexican population [16,17,18,37].

VD concentrations depend on many factors, including environmental and genetics. Likewise, Forrest et al. [38], showed that Hispanic women were 3.2 more likely to have VD deficiency than Caucasian women. Furthermore, having no college education, being obese, aging and no daily milk consumption are some of the factors also related to VD deficiency in addition to ethnicity. In our study, we found that women between 45 to 59 years and 60 to 74 years had a frequency of VD deficiency of 30% and 50%, respectively. Another factor associated with vitamin D deficiency is its intake. In our study, women with VD deficiency had a lower intake. However, these findings do not agree with the results from Contreras-Manzano et al. [37]. They did not observe differences in average VD consumption between the group with and without VD deficiency. Possible explanations for their findings could be the age of the women evaluated, and the food-frequency questionnaire used. Another variable frequently associated with VD status is the season of the year when evaluations are performed [40]. However, in this sample of Mexican postmenopausal women, there was no relationship between these two variables. This may be explained by the geographic situation of Mexico. The tropic of Cancer crosses above the central region of the country; therefore, changes in sunlight hours between summer and winter are minimal. This effect is especially important in the central and south regions.

An important finding was that women with VD deficiency had higher levels of fasting serum glucose and a higher prevalence of type 2 diabetes. VD has been previously related to insulin resistance and type 2 diabetes in observational studies [9,38]. Carrillo-Vega, et al. [9] observed that increased levels of glycated hemoglobin were associated with an OR of 1.16 (95% CI: 1.07–1.25) for VD deficiency in older Mexican adults. Although in this study, we only have data on glucose levels and self-reports of type 2 diabetes, in other studies these parameters are frequently used for the diagnosis of type 2 diabetes [43]. Our findings corroborate the association between type 2 diabetes and VD deficiency (OR 1.94 95% CI 1.27–2.94, data not shown).

To the best of our knowledge, this is the first study identifying the association of *GC* and *VDR* variants with VD deficiency in Mexican postmenopausal women. Vitamin D-binding protein (VDBP) is encoded by the *GC* gene. It binds to VD sterol metabolites for transporting them through the circulatory system towards target organs. Previous studies have demonstrated that the concentration of VD metabolites was strong and positively associated with serum VDBP levels [11,44,45,46]. In this study, the tag SNP rs2282679 on *GC* was associated with VD deficiency. Furthermore, we found almost a two fold risk increment for having VD deficiency in women who were homozygous for the G allele in the *GC* polymorphism rs2282679. Our data resembles previous associations of these SNPs through genome-wide association studies [11,31,47,48] in other ethnic populations, such as the Chinese population, where rs2298849 in the *GC* gene was significantly associated with serum 25(OH)D_3_ levels in postmenopausal women [33]. In addition, a positive association between plasma VDBP (Vitamin D Binding Protein) and 1,25(OH)_2_D concentration has been reported in a cohort from Denmark [49]. A recent report in non-Hispanic populations (White and Black), showed evidence that polymorphisms on the *GC* gene may be related to 25(OH)D status during pregnancy. The minor allele of the rs7041 SNP was related to increased 25(OH)D and the rs4588 SNP was associated with decreased 25(OH)D among pregnant women [50]. Although in our study, these polymorphisms were not observed to be associated with vitamin D levels, we cannot rule out the influence on VDBP levels in our population.

The *VDR* polymorphisms have been previously associated to VD status. Vitamin D receptor binds to 1-25-(OH)_2_D_3_, allowing transcription of genes that modulate VD metabolism [51]. A study in Hispanic individuals observed an inverse association between a *VDR* SNP (rs10783219) and 25(OH)D concentrations adjusted for age and gender (*p* = 0.004) [48]. Our results differ from Engelman et al. [30], as we did not observe an association between rs10783219 and VD deficiency in Mexican postmenopausal women. In contrast, an association was observed for the SNP rs4516035 (*p* = 0.012). This variant is located at a GATA binding site. The C allele is known to eliminate the GATA binding site, resulting in lower *VDR* promoter activity [52]. This observation leads us to postulate that the effect of some variants among the Mexican Mestizo population may be more significant than in Europeans. Additional studies in other populations are need to explore the extent of the association between these variants and VD deficiency.

We did not find significant association between variants in *CYP2R1, CYP27B1, CYP24A1* and *NADSYN1/DHCR7* genes and VD serum concentrations, while several studies have claimed to find association [52,53,54]. The negative results might be mainly attributed to ethnic differences between the Mexican population and other groups around the world. Additionally, this might be explained by the lacking functionality of these SNPs in Mexican populations.

On the other hand, studies regarding the relationship between *VDR* polymorphisms and BMD are inconclusive. A meta-analysis considering different populations showed an association between some *VDR* polymorphisms and low BMD [55]. The *VDR* ApaI polymorphism decreased the risk of osteoporosis in Caucasian postmenopausal women, while in Asian populations, *VDR* BsmI and *VDR* FokI were associated with an increased risk of osteoporosis. In this study, *VDR* gene polymorphisms (ApaI, BsmI, Cdx2, FokI and TaqI) were not analyzed. However, an association of the *NADSYN1/DHCR7* gene with osteopenia/osteoporosis was identified. This gene encodes the 7-dehydrocholesterol reductase, which catalyzes the production of cholesterol from 7-dehydrocholesterol, using NADPH, in the de novo synthesis pathway of 25(OH)D in the skin. More studies in Mexican population with other polymorphisms on more genes involved in VD metabolism are needed to confirm or rule out the association with bone mineral density observed in other populations.

The highest frequency (29%) of the rs4516035-C allele observed among ethnic groups from the South East (SE) of Mexico agrees with previous studies, which have reported that Amerindian ancestry shows an increasing gradient from North to South. These findings contrast markedly with the distribution of VD deficiency along the country reported in the National Health and Nutrition Survey-2012 (ENSANUT-2012). The ENSANUT observed that the frequency of VD deficiency was higher in indigenous women from the South region of the country when compared to those from the North [8,38]. We hypothesized that the highest VD deficiency frequencies observed in the South of the country may be related to a genetic predisposition, low dietary intake of VD and the more intense pigmentation of the skin.

This study has some limitations that should be considered. First, it was based on a cross-sectional design and the analyzed SNPs were selected from previous reports conducted in Hispanic and European populations [11,30,31]. Second, it does not include measurements of 25(OH)D in Amerindian groups or sun exposure in Mexican postmenopausal women. However, we use the blood collection season as a proxy for sun exposure. Third, the women in the present study are part of a cohort study that included health workers of the Mexican Institute of Social Security, who are likely more educated and healthier than the general population of Mexico. Therefore, our findings related to sociodemographic and dietary variables cannot predict the association at the national level. However, we consider that the HWCS population could be representative of adults living in urban areas in Central Mexico. Fourth, after adjusting our analyses for multiple testing, no variant remained associated with VD deficiency, probably due to our limited sample size.

## 5. Conclusions

In conclusion, this is the first study of genetic variants related to VD deficiency in the Mexican population. We show that polymorphisms on the *VDR* and *GC* genes are associated with VD deficiency in postmenopausal women. These results suggest an important role for genetic variants in the high prevalence of VD deficiency observed in a sunny country with a strong indigenous ethnic background, such as Mexico.

## Figures and Tables

**Table 1:** Characteristics of postmenopausal women of the Health Workers Cohort Study (2010).

Characteristics	*n* = 689
Age (years) *	62.2 (8.8)
45−59, *n* (%)	279 (40.9)
60−74, *n* (%)	340 (49.9)
>74, *n* (%)	63 (9.2)
BMI (kg/m^2^) *	28.1 (4.7)
Nutritional Status, *n* (%)	
Overweight, λ	306 (44.9)
Obesity, λ	204 (29.9)
Waist circumference (cm) *	95.9 (11.3)
Body fat proportion *	45.6 (6.1)
Leisure time physical activity (min/day) *	20.9 (27.3)
Active (≥30 min/day), %	186 (27.3)
Serum 25(OH)D (ng/mL) *	21.0 (5.7)
25(OH)D Deficiency (<20ng/mL), *n* (%)	298 (43.8)
Bone Femoral neck Density (g/cm^2^) *	0.87 (0.13)
Bone Femoral neck status, *n* (%)	
Osteopenia	224 (32.8)
Osteoporosis	32 (4.7)
Bone Spine Density (g/cm^2^) *	1.00 (0.16)
Bone Spine status, *n* (%)	
Osteopenia	324 (47.5)
Osteoporosis	165 (24.2)
Season of blood collection, *n* (%)	
Winter	112 (16.4)
Spring	412 (60.4)
Summer	113 (16.6)
Autumn	45 (6.6)
Diet	
Total energy intake (kcal/day) *	1805 (854)
Alcohol (g/day) *	1.6 (4.2)
Vitamin D intake (IU/day) *	199 (157)
Calcium intake (mg/day) *	918 (545)
Calcium supplements, *n* (%)	194 (28.5)
Medication use, *n* (%)	
Hormone replacement therapy, *n* (%)	44 (6.5)

* Mean (standard deviation). λ Overweight (25 to <30 kg/m^2^) and Obesity (≥30 kg/m^2^).

**Table 2:** Characteristics of postmenopausal women of the Health Workers Cohort Study by vitamin D status (2010).

	Non-Deficient: 25-OH-D ≥20 ng/mL *n* = 386 (56.2%)	Deficient: 25-OH-D <20 ng/mL *n* = 301 (43.8%)	*p* Value
Age(years) *	61.2 (8.4)	63.4 (9.2)	0.0008
Age categories, *n* (%)			
45–59	180 (46.6)	101 (33.6)	0.006
60–74	179 (46.4)	163 (54.2)	0.04
>74	27 (7.0)	37 (12.3)	0.02
BMI (kg/m^2^) *	28.1 (4.7)	28.2 (4.8)	0.76
Nutritional Status, *n* (%)			
Overweight, λ	175 (45.3)	133 (44.2)	0.71
Obesity, λ	114 (29.5)	91 (30.2)	0.84
Waist circumference (cm) *	95.5 (11.1)	96.4 (11.6)	0.30
Body fat proportion *	45.7 (6.2)	45.4 (6.1)	0.53
Leisure time physical activity (min/day) *	20.5 (27.7)	19.4 (26.0)	0.60
Active (≥30 min/day), %	28.5	25.8	0.43
Femoral neck (g/cm^2^) *	0.89 (0.13)	0.86 (0.13)	0.004
Femoral status, *n* (%)			
Osteopenia	121 (31.4)	107 (35.7)	0.23
Osteoporosis	17 (4.4)	16 (5.3)	0.57
Spine BMD (g/cm^2^) *	1.01 (0.16)	0.99 (0.16)	0.004
Spine status, *n* (%)			
Osteopenia	187 (48.6)	139 (46.3)	0.55
Osteoporosis	83 (21.6)	84 (28.0)	0.05
Season of blood collection, *n* (%)			
Winter	54 (14.8)	55 (18.3)	0.22
Spring	249 (64.5)	163 (54.1)	0.006
Summer	58 (15.0)	61 (20.3)	0.07
Autumn	22 (5.7)	22 (7.3)	0.40
Diet			
Total energy intake (kcal/day) *	1807 (826)	1814 (895)	0.92
Alcohol intake (g/day) *	1.5 (3.5)	1.8 (4.9)	0.28
Vitamin D intake (IU/day) *	209 (160)	188 (151)	0.02
Calcium intake (mg/day) *	941 (537)	890 (554)	0.22
Calcium supplements, *n* (%)	116 (30.1)	79 (26.3)	0.29
Medication use			
Hormone replacement therapy, *n* (%)	23 (6.0)	21 (7.0)	0.60
Calcium, *n* (%)	9 (2.3)	5 (1.7)	0.55

* Mean (standard deviation). *p* values from *t*-test (continuous variables) or chi^2^ test (categorical variables). λ Overweight (25 to <30 kg/m^2^) and Obesity (≥ 30 kg/m^2^).

**Table 3:** Odds ratios for the association of vitamin D metabolism genetic variants and vitamin D deficiency in a sample of 689 postmenopausal women.

Gene	SNP	Genotype	Not Deficient * *n* (%)	Deficient * *n* (%)	Unadjuste d OR (CI 95%)	*p* Value	Adjusted Model *OR* (CI 95%)	*p* Value
*VDR*	rs10783219	TT	135 (35.3)	94 (31.5)	1.0		1.0	
		TA	170 (44.5)	145 (48.7)	1.22 (0.87–1.73)	0.248	1.20 (0.84–1.72)	0.315
		AA	77 (20.2)	59 (19.8)	1.10 (0.72–1.69)	0.662	1.13 (0.72–1.75)	0.597
		Additive model			1.07 (0.87–1.32)	0.537	1.10 (0.89–1.38)	0.377
*VDR*	rs4516035	TT	219 (57.5)	146 (49.0)	1.0		1.0	
		TC	141 (37.0)	130 (43.6)	1.38 (1.01–1.90)	0.045	1.41 (1.01–1.95)	0.041
		CC	21 (5.5)	22 (7.4)	1.57 (0.83–2.96)	0.162	1.75 (0.91–3.35)	0.091
		Additive model			1.31 (1.02–1.68)	0.032	1.40 (1.08–4.43)	0.012
*GC*	rs2282679	TT	247 (64.6)	164 (55.0)	1.0		1.0	
		TG	127 (33.3)	119 (39.9)	1.41 (1.03–1.94)	0.034	1.48 (1.07–2.06)	0.019
		GG	8 (2.1)	15 (2.0)	2.82 (1.17–6.81)	0.021	3.00 (1.20–7.49)	0.019
		Additive model			1.50 (1.14–1.97)	0.003	1.53 (1.15–2.04)	0.003

* Non-deficient: serum 25(OH)D ≥20 ng/mL; deficient: serum 25(OH)D <20 ng/mL. Adjusted model: adjusted for age (<60, 60–74, >74 years), body fat proportion (tertiles), vitamin D intake IU/day (tertiles), season of blood collection (winter, spring, summer, autumn) and leisure time physical activity (>30 min/day).

**Table 4:** Genotype frequencies of *GC* and *VDR* variants in 37 Mexican Amerindian groups.

Populations		Genotypic Frequencies											
Geographical Region	Ethnic Group	rs2298850 (Proxy for rs2282679)						rs4516035					
		GG		GC		CC		TT		CT		CC	
		Count	Freq	Count	Freq	Count	Freq	Count	Freq	Count	Freq	Count	Freq
North (N)	Guarijio	2	0.22	4	0.44	3	0.33	8	0.89	1	0.11	0	0
	Mayo	4	0.80	1	0.20	0	0	3	0.60	2	0.40	0	0
	Seri	7	1.0	0	0	0	0	8	1.0	0	0	0	0
	Tarahumara	19	0.56	15	0.44	0	0	33	0.97	1	0.03	0	0
	Yaqui	4	1.0	0	0	0	0	3	0.75	1	0.25	0	0
	Total	36	0.72	19	0.22	3	0.067	55	0.84	5	0.16	0	0
Central West (CW)	Purepecha	7	0.78	2	0.22	0	0	7	0.78	2	0.22	0	0
	Total	7	0.78	2	0.22	0	0	7	0.78	2	0.22	0	0
Central East (CE)	Huasteco	7	0.70	3	0.30	0	0	7	0.70	3	0.30	0	0
	Matlaltzinca	8	0.80	1	0.10	1	0.10	6	0.60	4	0.40	0	0
	Mazateco PUE	4	0.44	5	0.56	0	0	4	0.44	5	0.56	0	0
	Nahuatl MOR	8	0.89	1	0.11	0	0	0	0	8	0.89	1	0.11
	Nahuatl PUE	5	0.71	2	0.29	0	0	4	0.57	3	0.43	0	0
	Nahuatl SLP	6	1.0	0	0	0	0	4	0.67	2	0.33	0	0
	Otomi	28	0.78	8	0.22	0	0	22	0.61	11	0.31	3	0.08
	Pame	8	0.89	1	0.11	0	0	7	0.70	3	0.30	0	0
	Popoluca de la Sierra	7	0.88	1	0.12	0	0	7	0.88	1	0.12	0	0
	Tlahuica	5	1.0	0	0	0	0	5	1.0	0	0	0	0
	Totonaco PUE	6	0.55	5	0.45	0	0	6	0.55	5	0.45	0	0
	Totonaco VER	7	0.70	3	0.30	0	0	6	0.60	4	0.40	0	0
	Total	99	0.79	30	0.206	1	0.009	78	0.601	49	0.381	4	0.018
South (S)	Chinanteco	5	0.50	5	0.50	0	0	7	0.70	3	0.30	0	0
	Chontal OAX	3	0.50	2	0.33	1	0.17	3	0.50	2	0.33	1	0.17
	Huave	6	0.86	1	0.14	0	0	5	0.72	1	0.14	1	0.14
	Mazateco OAX	4	0.57	2	0.29	1	0.14	5	0.71	2	0.29	0	0
	Mixe	8	0.89	1	0.1	0	0	4	0.44	5	0.56	0	0
	Mixteco Alto	8	0.73	3	0.27	0	0	6	0.55	5	0.45	0	0
	Mixteco Costa	5	0.50	4	0.40	1	0.10	7	0.70	3	0.30	0	0
	Zapoteco	6	0.75	2	0.25	0	0	3	0.38	2	0.24	3	0.38
	Total	45	0.66	20	0.29	3	0.05	40	0.587	23	0.328	5	0.086
South East (SE)	Chuj	5	0.83	1	0.17	0	0	2	0.33	2	0.33	2	0.33
	Jakalteco	3	0.60	2	0.40	0	0	2	0.40	3	0.60	0	0
	Kanjobal	9	0.90	1	0.10	0	0	2	0.20	7	0.70	1	0.10
	Kaqchikel	6	0.67	3	0.33	0	0	5	0.56	3	0.33	1	0.11
	Mam	4	0.44	4	0.44	1	0.12	7	0.78	2	0.22	0	0
	Maya	12	0.80	3	0.20	0	0	9	0.60	4	0.27	2	0.13
	Mocho	5	0.83	1	0.17	0	0	5	0.83	0	0	1	0.17
	Tojolabal	5	0.63	3	0.37	0	0	5	0.63	3	0.37	0	0
	Tseltal	3	0.60	2	0.40	0	0	1	0.20	3	0.60	1	0.20
	Tsotsil	5	0.63	3	0.37	0	0	6	0.75	2	0.25	0	0
	Zoque	3	0.67	2	0.33	0	0	4	0.80	1	0.20	0	0
	Total	60	0.68	25	0.31	1	0.01	48	0.552	30	0.353	8	0.095

Mexico State; MOR, Morelos; OAX, Oaxaca; PUE, Puebla; SLP, San Luis Potosi; VER, Veracruz.

## Supplementary Materials

The following are available online at http://www.mdpi.com/2072-6643/10/9/1175/s1, Table S1: Allele frequencies of SNPs lying on selected genes involved in the metabolism of vitamin D, Table S2: Multiple logistic regressions for Vitamin D deficiency as the dependent variable, Table S3: Odds ratio for the association of vitamin D metabolism genetic variants and vitamin D deficiency in a sample of 400 postmenopausal women. Table S4: Multivariate ORs and 95% CIs for the association between *DHCR7/NADSYN1* tag SNP and osteopenia/osteoporosis (*n* = 689), Figure S1: Flowchart of study population, Figure S2: Linkage disequilibrium map of single nucleotide polymorphisms in the *VDR* and GC genes.
