# Supplementary material for: Association between Vitamin D Deficiency and Single Nucleotide Polymorphisms in the Vitamin D Receptor and GC Genes and Analysis of Their Distribution in Mexican Postmenopausal Women
Source: Nutrients. 2018 Aug 27;10(9):1175. doi: 10.3390/nu10091175 (PMC6164456; doi:10.3390/nu10091175)

Supplemental Tables and figures

Table S1. Allele frequencies of SNPs lying on selected genes involved in the metabolism of vitamin D.

No.	Gene	SNP	Minor Allele	Major Allele	Location	Minor Allele Frequency CEU[1]	MXL[2]	HWCS[3]
1	*PDE3B*	rs7116978	T	C	14838347	37	38	36.5
2	*CYP2R1*	rs1993116	C	T	14866810	59	62	37.4
3	--	rs12794714	A	G	14870151	43	51	44.2
4	*CYP2R1*	rs10741657	A	G	14871454	40	38	37.4
5	*CYP2R1*	rs2060793	G	A	14871886	60	60	37.7
6	*CYP2R1*	rs10766197	A	G	14878456	46	33	25.6
7	*VDR*	rs10783219	A	T	46581755	64	44	42.7
8	*VDR*	rs4516035	C	T	46586093	37	27	26.7
9	*VDR*	rs7139166	G	C	46586601	37	27	26.5
10	*CYP24A1*	rs2244719	T	C	52216265	60	48	40.2
11	*CYP24A1*	rs2296241	A	G	52219626	54	33	39.5
12	*CYP27B1*	rs4646536	G	A	56444255	33	34	36.8
13	*CYP27B1*	rs703842	G	A	56449006	33	34	36.9
14	*DHCR7*	rs1790349	C	T	70819998	19	16	16.1
15	*NADSYN1*	rs7944926	G	A	70843273	70	47	46.8
16	*NADSYN1*	rs4944957	G	A	70845683	70	47.7	47.7
17	*NADSYN1*	rs12800438	A	G	70848651	70	47.7	46.1
18	*NADSYN1*	rs3794060	T	C	70865327	70	47	45.4
19	*NADSYN1*	rs3829251	A	G	70872207	19	20	19.4
20	*LOC645474*	rs4945008	G	A	70898896	70	47	46.4
21	*LOC645474*	rs11234027	A	G	70911755	20	20	16.5
22	*GC*	rs12512631	C	T	72820195	35	31	41.1
23	*GC*	rs17467825	G	A	72824381	26	30	23.9
24	*GC*	rs2282679	G	T	72827247	24	30	23.9
25	*GC*	rs3755967	T	C	72828262	26	30	24
26	*GC*	rs2298850	C	G	72833131	23	27	22.9
27	*GC*	rs7041	C	A	72837198	57	45	47.6
28	*GC*	rs1155563	C	T	72862352	27	27	22.3
29	*C10orf88*	rs6599638	A	G	124694139	49	31	34.4

[1]Utah residents with Northern and Western European ancestry (CEU) obtained from 1000 Genomes. [2]Mexican ancestry in Los Angeles, California (MXL) obtained from 1000 Genomes. [3]Postmenopausal women of Health Workers Cohort Study (HWCS). SNPs with p-value >0.05 of the chi-square test for Hardy-Weinberg equilibrium.

Table S2. Multiple logistic regressions for Vitamin D deficiency as the dependent variable.

Variables	OR (95% CI)	*p* Value
Age	1.04 (1.02–1.06)	<0.001
45-59 years	1.0	
60-74 years	1.95 (1.36–2.79)	<0.001
>74 years	3.07 (1.70–5.56)	<0.001
Education		
Elementary/Secondary	1.0	
High school	1.15(0.72–1.84)	0.560

University or more	1.30 (0.89–1.93)	0.173
Non smoker	1.0	
Smoking Current	1.59 (0.84–3.03)	0.155
Past	0.80 (0.55–1.16)	0.244
Vitamin D intake (UI)		
Tertile 1	1.0	
Tertile 2	0.79 (0.53–1.16)	0.229
Tertile 3	0.61 (0.39–0.95)	0.028
Season of blood collection		
Winter	1.0	
Spring	0.62 (0.40–0.96)	0.032
Summer	0.99 (0.57–1.70)	0.968
Autunm	0.99 (0.48–2.02)	0.973
Leisure time physical activity (min/day)		
Active (≥30 min/day)	0.91 (0.63–1.30)	0.597
BMI, Normal		
Overweight	0.98 (0.66–1.45)	0.913
Obesity	1.08 (0.70–1.66)	0.725
Body fat proportion	1.00 (0.97–1.04)	0.829
Tertile 1	1.0	
Tertile 2	1.29 (0.87–1.91)	0.203
Tertile 3	0.95 (0.64–1.41)	0.792

Multivariate models.

Table S3: Odds ratio for the association of vitamin D metabolism genetic variants and vitamin D deficiency in a sample of 400 postmenopausal women.

	Gene	SNP	Genotypes	Not Deficient*, n (%)	Deficient*, n (%)	Crude OR(CI 95%)	P value	Model OR(CI 95%)	P value
1	*VDR*	rs10783219	TT	94 (38.5)	39 (26.5)	1.0		1.0	
			TA	105 (43.0)	76 (51.7)	1.74(1.08-2.81)	0.022	1.72(1.04-2.87)	0.036
			AA	45 (18.4)	32 (21.8)	1.71(0.95-3.08)	0.072	1.89(1.02-3.53)	0.045
		Additive model				1.34(1.01-1.79)	0.042	1.40(1.03-1.90)	0.031
2	*VDR*	rs4516035	TT	146 (60.1)	61 (41.5)	1.0		1.0	
			TC	86 (35.4)	72 (49.0)	2.00(1.30-3.09)	0.002	2.00(1.27-3.17)	0.003
			CC	11 (4.5)	14 (9.5)	3.05(1.30-7.09)	0.01	3.27(1.35-7.96)	0.009
		Additive model				1.87(1.33-2.62)	<0.001	1.89(1.32-2.70)	0.001
3	*VDR*	rs7139166	CC	149 (60.8)	61 (41.5)	1.0		1.0	
			CG	84 (34.3)	72 (49.0)	2.09(1.36-3.23)	0.001	2.14(1.35-3.40)	0.001
			GG	12 (4.9)	14 (9.5)	2.85(1.25-6.51)	0.013	3.11(1.30-7.44)	0.011
		Additive model				1.87(1.34-2.61)	<0.001	1.93(1.35-2.75)	<0.001
4	*GC*	rs12512631	TT	77 (31.4)	56 (38.1)	1.0		1.0	
			TC	121 (49.4)	74 (50.3)	0.84(0.54-1.32)	0.45	0.89(0.56-1.43)	0.63
			CC	47 (19.2)	17 (11.6)	0.50(0.26-0.96	0.036	0.59(0.30-1.16)	0.129
		Additive model				0.74(0.54-0.99)	0.047	0.80(0.58-1.10)	0.165
5	*GC*	rs17467825	AA	150 (61.5)	72 (49.0)	1.0		1.0	
			AG	87 (35.7)	64 (43.5)	1.53(1.00-2.35)	0.051	1.64(1.04-2.60)	0.033
			GG	7 (2.9)	11 (7.5)	3.27(1.22-8.80)	0.019	3.48(1.19-10.20)	0.023
		Additive model				1.64(1.16-2.33)	0.005	1.73(1.19-2.52)	0.004
6	*GC*	rs2282679	TT	150 (61.5)	72 (49.0)	1.0		1.0	
			TG	87 (35.7)	64 (43.5)	1.53(1.00-2.35)	0.051	1.65(1.04-2.61)	0.032
			GG	7 (2.9)	11 (7.5)	3.27(1.22-8.80)	0.019	3.45(1.18-10.12)	0.024
		Additive model				1.64(1.16-2.33)	0.005	1.73(1.19-2.52)	0.004

7	*GC*	rs3755967	CC	150 (61.2)	72 (49.0)	1.0		1.0	
			CT	88 (35.9)	64 (43.5)	1.52(0.99-2.32)	0.057	1.61(1.02-2.54)	0.040
			TT	7 (2.9)	11 (7.5)	3.27(1.22-8.80)	0.019	3.48(1.19-10.18)	0.023
		Additive model				1.63(1.15-2.31)	0.005	1.71(1.18-2.49)	0.005
8	*GC*	rs2298850	GG	154 (62.9)	75 (51.0)	1.0		1.0	
			GC	84 (34.3)	62 (42.2)	1.52(0.99-2.33)	0.057	1.64(1.04-2.60)	0.034
			CC	7 (2.9)	10 (6.8)	2.93(1.07-8.01)	0.036	2.88(0.98-8.51)	0.055
		Additive model				1.59(1.12-2.27)	0.010	1.67(1.14-2.43)	0.008
9	*GC*	rs7041	AA	59 (24.1)	51 (34.7)	1.0		1.0	
			AC	123 (50.2)	67 (45.6)	0.63(0.39-1.02)	0.059	0.70(0.42-1.15)	0.156
			CC	63 (25.7)	29 (19.7)	0.53(0.30-0.95)	0.033	0.60(0.33-1.10)	0.099
		Additive model				0.72(0.54-0.96)	0.027	0.77(0.57-1.04)	0.087
10	*GC*	rs1155563	TT	155 (63.3)	72 (49.0)	1.0		1.0	
			TC	86 (35.1)	69 (46.9)	1.73(1.13-2.64)	0.011	1.76(1.13-2.76)	0.013
			CC	4 (1.6)	6 (4.1)	3.23(0.88-11.80)	0.076	2.96(0.71-12.34)	0.135
		Additive model				1.75(1.20-2.54)	0.004	1.76(1.18-2.61)	0.006

*Not deficient: Vitamin D >20 nmol/L; Deficient: Vitamin D <19.9 nmol/L. Adjusted model: adjusted for age (<60,60-74,>74 years), body fat proportion (tertiles), vitamin D intake (tertiles), season of blood collection (winter, spring, summer, autumn), leisure time physical activity (≥30 min) and ancestry (three factors).

Table S4: Multivariate ORs and 95% CIs for the association between *DHCR7/NADSYN1* tag SNP and osteopenia/osteoporosis (n=689).

SNP	Genotype	Normal	Osteopenia/ Osteoporosis	Crude model	P value	Model	P value
rs3794060	CC	130 (30.7)	59 (22.7)	1.0		1.0	
	CT	215 (50.8)	140 (53.9)	1.43(0.99-2.09)	0.059	1.57(1.03-2.40)	0.035
	TT	78 (18.4)	61 (23.5)	1.72(1.09-2.72)	0.020	1.83(1.10-3.06)	0.021
	Additive model			1.32(1.05-1.65)	0.016	1.36(1.06-1.76)	0.017
rs4944957	AA	125 (29.6)	58 (22.2)	1.0		1.0	
	AG	207 (48.9)	134 (51.3)	1.40(0.95-2.04)	0.086	1.58(1.03-2.42)	0.036
	GG	91 (21.5)	69 (26.4)	1.63(1.05-2.54)	0.029	1.77(1.08-2.91)	0.025
	Additive model			1.28(1.03-1.59)	0.028	1.33(1.04-1.71)	0.023

Adjusted model: adjusted for age (<60, 60-74,>74 years), and body fat proportion (tertiles).

Figure S1. Flowchart of study population.

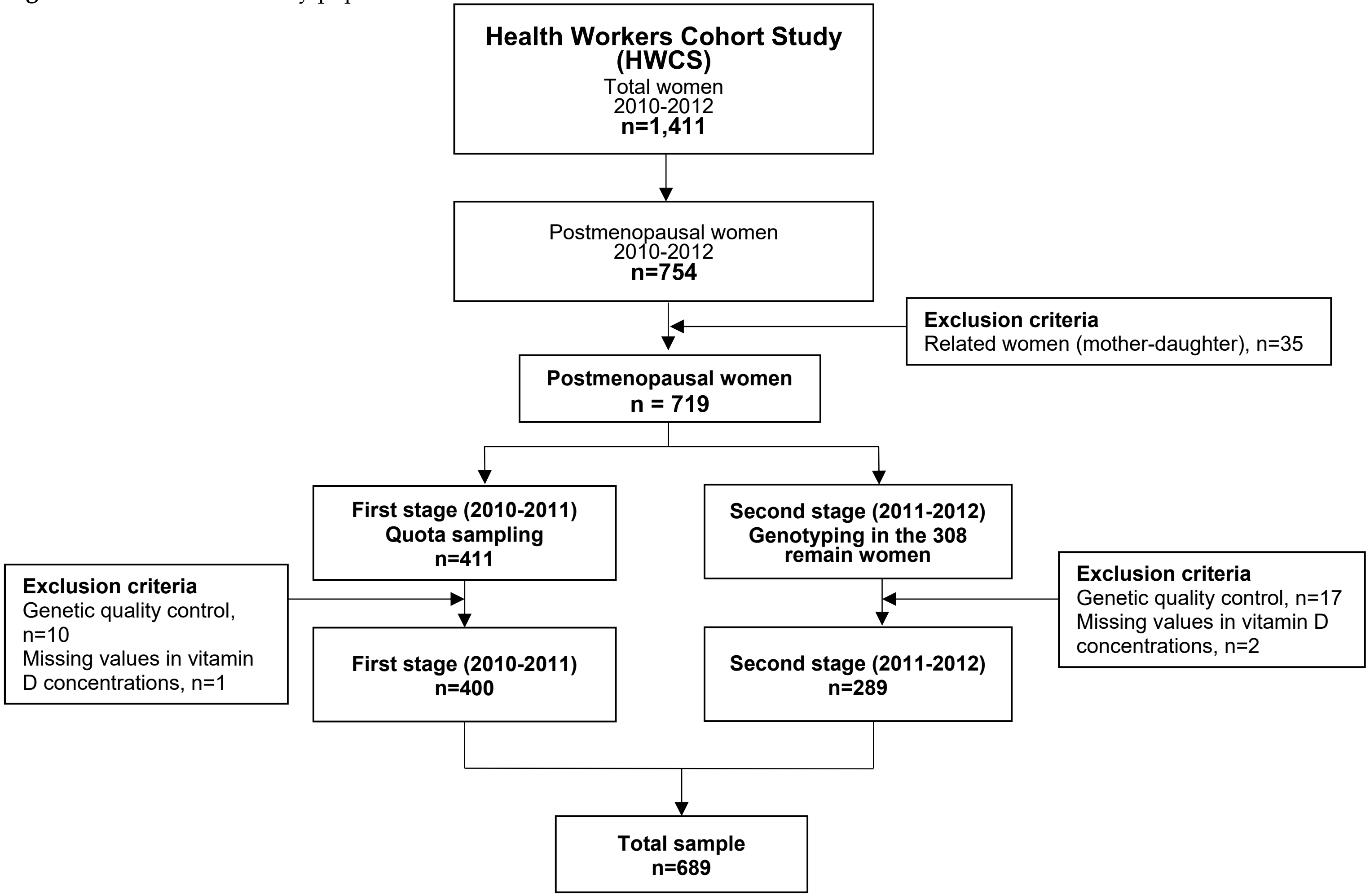

Figure S2: Linkage disequilibrium map of single nucleotide polymorphisms in the VDR and GC genes.

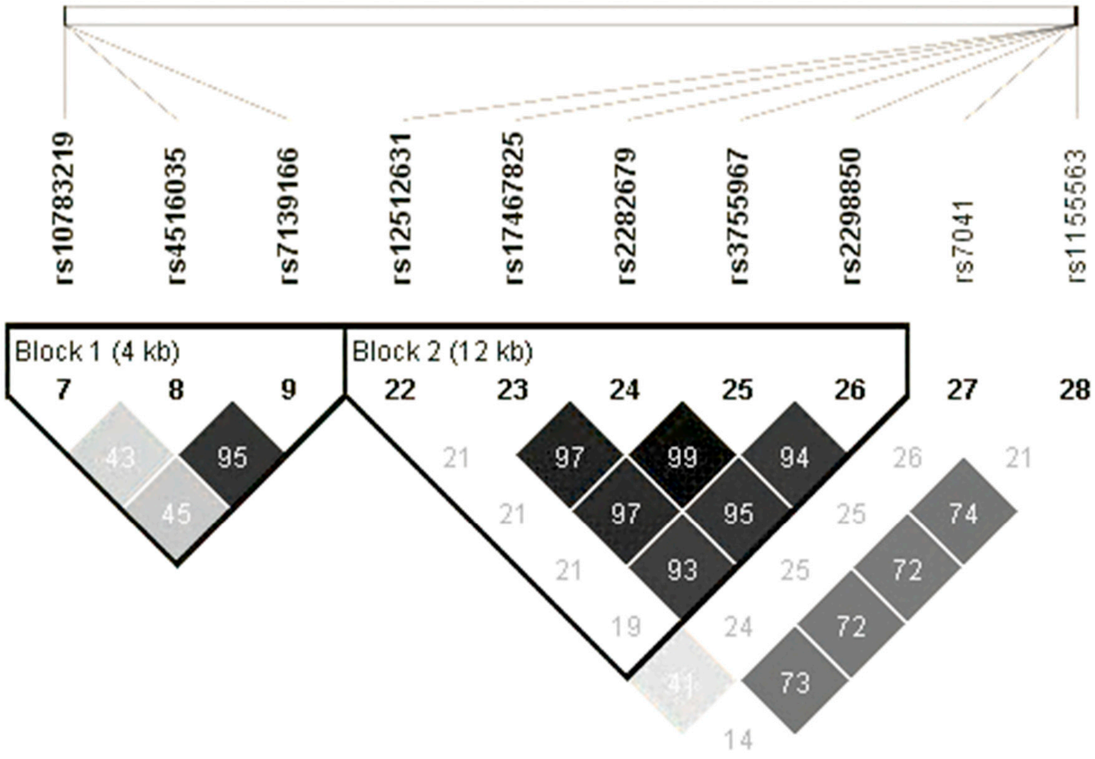

Supplement: Supplementary file 1 [file nutrients-10-01175-s001.pdf]
